# Thoracic laminectomy and midline myelotomy for resection of a spinal ependymoma

**DOI:** 10.3171/2023.6.FOCVID2386

**Published:** 2023-10-01

**Authors:** Lea Scherschinski, Ethan A. Winkler, Charuta G. Furey, Timothy C. Gooldy, Joshua S. Catapano, Michael T. Lawton

**Affiliations:** Department of Neurosurgery, Barrow Neurological Institute, St. Joseph’s Hospital and Medical Center, Phoenix, Arizona

**Keywords:** ependymoma, laminectomy, myelotomy, safe entry zone, spine

## Abstract

Spinal cord ependymomas comprise 25% of all intramedullary tumors and are typically treated with resection. A man in his mid-60s presented with imbalance and sensory deficits in both lower extremities, and a spinal thoracic intramedullary ependymoma spanning the levels T2 and T3 was diagnosed. After a laminectomy was performed, the tumor was microsurgically resected, and the patient demonstrated no neurological deficits on postoperative examination. Subsequent MRI showed complete resection of the tumor. This video showcases a thoracic intramedullary ependymoma resected using careful microdissection into the median raphe as a safe entry zone to preserve neurological function.

## Transcript

This video will demonstrate the thoracic laminectomy and midline myelotomy for resection of a spinal ependymoma.

### 0:29 Clinical Presentation.

The patient was in his mid-60s. He presented with several months’ history of imbalance and paresthesias in his legs. On examination, he had decreased sensation in his lower extremities.

### 0:42 Imaging Review.

Here is his axial and sagittal MRI, demonstrating intradural intramedullary contrast-enhancing mass from T2 to T3 shown at the arrow. This was most consistent with ependymoma.

### 1:05 Surgical Strategy.

A 2-level laminectomy was planned using intraoperative X-ray localization. Key surgical steps consisted of a T2 to T3 laminectomy, midline durotomy with wide arachnoidal dissection, microdissection into the median raphe and a midline myelotomy, drainage of the cystic component of the tumor, circumferential dissection around the tumor, piecemeal resection, and, finally, hemostasis.

### 1:29 Beginning of Operative Video.

Here is a view of the patient’s spinal cord. The head is to your left and the feet are to your right.

### 1:36 Midline Myelotomy.

First, the midline vein on the dorsal surface of the spinal cord is mobilized to the patient’s right side. This reveals the midline raphe. I like to do this with sharp dissection, and these little perforating arteries can be found, which travel down the raphe and help guide the dissection into the right plane. Now these bypass forceps are used to help separate the dorsal columns. These instruments have a very fine tip and just the right spreading force to separate the dorsal columns.

### 2:14 Identification and Exposure of the Tumor.

You can see, at the depths, I have reached the capsule of the tumor. There is a color change, and the tissue is grayish in its color. Stitches are now being placed in the pia in order to provide some very gentle traction on the 2 hemicords. This helps to hold the hemicords apart and widen the surgical corridor into the tumor. It is important to extend the midline myelotomy to the superior and inferior poles of the tumor. Otherwise, you create a ledge, where it is difficult to see around the poles of the tumor. So, before I attack the tumor, I am making sure that I extended that myelotomy sufficiently.

### 3:01 Circumferential Dissection and Removal of the Tumor.

The tumor is now in plain view. It had a large cystic core, and the cystic fluid has been largely evacuated here. I am placing another pial stitch for additional traction on the hemicord. Now the plane of dissection around the tumor can be worked. Notice how the tissue itself is slightly grayish in color, and the normal spinal cord has a yellowish gliotic tint. This helps to identify the separation plane. This sharp round knife is very useful in developing that separation plane. The closed tips with my microscissors are also a nice way to develop this plane. Sharp dissection is again utilized around the margin of the tumor to get every last bit of the tumor separated from the cord. By sharply dissecting and teasing it away, I am slowly working that plane. Notice that I am doing circumferential dissection, moving from one side to the next. Now the tumor is largely freed, so that I can remove these liberated chunks of tumor with my grasping forceps. With some gentle traction, I am able to peel the remaining adhesions apart. The tumor comes out nicely. The tumor was largely cystic, and the solid component is small.

### 4:37 Overview of the Exposure and Closure.

Once the cavity has been completely cleaned out, these stay sutures are cut and released. The arachnoid is pulled back together over the surface of the spinal cord to prevent any delayed tethering.

### 4:51 Disease Background.

Spinal cord ependymomas account for approximately a quarter of intramedullary tumors of the spinal cord and about 2% of CNS malignancies.^1–3^ Classic ependymomas are WHO grade II tumors and often have this cystic component.^4,5^ These illustrations show the midline dorsal raphe, which acts as a safe entry zone through the spinal cord to the tumor, which arises from that central canal.^6^ The midline myelotomy must travel all the way down to the center of the cord.

### 5:25 Clinical Outcome.

The patient tolerated the procedure well with no new neurological deficits. He was discharged home on postoperative day 5.

### 5:33 Postoperative Imaging.

His postoperative MRI, shown here, demonstrates complete resection of the pathology.

### 5:40 Conclusions.

In conclusion, microsurgical resection remains the gold-standard treatment for spinal cord ependymomas. Microdissection identifies the median raphe and follows perforating arteries down this raphe to complete the midline myelotomy and minimize iatrogenic injury to the dorsal columns. Use of bypass forceps, with its fine tips and gentle spreading force, increases the precision of this critical midline myelotomy. Meticulous dissection of the surgical plane surrounding the tumor helps to achieve complete microsurgical dissection. Thank you.

## Acknowledgments

We thank the staff of Neuroscience Publications at Barrow Neurological Institute for assistance with manuscript and video preparation.

## Disclosures

The authors report no conflict of interest concerning the materials or methods used in this study or the findings specified in this publication.

## Author Contributions

Primary surgeon: Lawton. Assistant surgeon: Winkler, Catapano. Editing and drafting the video and abstract: Lawton, Scherschinski, Winkler. Critically revising the work: all authors. Reviewed submitted version of the work: all authors. Approved the final version of the work on behalf of all authors: Lawton. Supervision: Lawton.

## Supplemental Information

### Patient Informed Consent

The necessary patient informed consent was obtained in this study.
